# Alpha_1_-adrenergic receptor blockade in the ventral tegmental area attenuates acquisition of cocaine-induced pavlovian associative learning

**DOI:** 10.3389/fnbeh.2022.969104

**Published:** 2022-08-04

**Authors:** Wojciech B. Solecki, Michał Kielbinski, Joanna Bernacka, Katarzyna Gralec, Adam Klasa, Kamil Pradel, Karolina Rojek-Sito, Ryszard Przewłocki

**Affiliations:** ^1^Department of Neurobiology and Neuropsychology, Institute of Applied Psychology, Jagiellonian University, Kraków, Poland; ^2^Laboratory of Pharmacology and Brain Biostructure, Department of Pharmacology, Maj Institute of Pharmacology, Polish Academy of Sciences, Kraków, Poland; ^3^Department of Molecular Neuropharmacology, Maj Institute of Pharmacology, Polish Academy of Sciences, Kraków, Poland; ^4^Department of Neurophysiology and Chronobiology, Institute of Zoology and Biomedical Research, Jagiellonian University, Kraków, Poland; ^5^Department of Behavioral Neuroscience and Drug Development, Maj Institute of Pharmacology, Polish Academy of Sciences, Kraków, Poland

**Keywords:** ventral tegmental area (VTA), phasic dopamine, alpha_1_-adrenergic receptor, Pavlovian associative learning, salience

## Abstract

Activity of the alpha_1_-adrenergic receptor (α_1_-AR) in the ventral tegmental area (VTA) modulates dopaminergic activity, implying its modulatory role in the behavioral functions of the dopamine (DA) system. Indeed, intra-VTA α_1_-AR blockade attenuates conditioned stimulus dependent behaviors such as drug seeking responses signifying a role of the noradrenergic signaling in the VTA in conditioned behaviors. Importantly, the role of the VTA α_1_-AR activity in Pavlovian associative learning with positive outcomes remains unknown. Here, we aimed to examine how intra-VTA α_1_-AR blockade affects acquisition of cocaine-induced Pavlovian associative learning in the conditioned place preference (CPP) paradigm. The impact of α_1_-AR blockade on cocaine-reinforced operant responding and cocaine-evoked ultrasonic vocalizations (USVs) was also studied. In addition, both α_1_-AR immunoreactivity in the VTA and its role in phasic DA release in the nucleus accumbens (NAc) were assessed. We demonstrated cellular localization of α_1_-AR expression in the VTA, providing a neuroanatomical substrate for the α_1_-AR mechanism. We showed that prazosin (α_1_-AR selective antagonist; 1 μg/0.5 μl) microinfusion attenuated electrically evoked DA transients in the NAc and dose-dependently (0.1–1 μg/0.5 μl) prevented the acquisition of cocaine CPP but did not affect cocaine-reinforced operant responding nor cocaine-induced positive affective state (measured as USVs). We propose that the VTA α_1_-AR signaling is necessary for the acquisition of Pavlovian associative learning but does not encode hedonic value. Thus, α_1_-AR signaling in the VTA might underlie salience encoding of environmental stimuli and reflect an ability of alerting/orienting functions, originating from bottom-up information processing to guide behaviors.

## Introduction

Phasic DA release in the forebrain is crucial for both the acquisition of Pavlovian association learning and the expression of conditioned behaviors. In particular, increases in phasic DA responses to unpredicted rewards are necessary for the acquisition of reward-based learning, allowing an organism to predict the value of an upcoming event and adequately guide its behavior in the future (Glimcher, 2011). Accordingly, attenuation of burst activity of the DA neurons might impair acquisition of reward-based associative learning (Zweifel et al., 2009).

There is mounting evidence for modulatory inputs from noradrenergic (NA) structures into the ventral tegmental area (VTA) in shaping moment-to-moment attention and goal-orienting behavior (Sara and Bouret, 2012; Bouret and Richmond, 2015; Jahn et al., 2018). Neurochemical and neurophysiological studies have since identified adrenergic receptors on both DA projection neurons and GABA interneurons as well as both excitatory and inhibitory afferent terminals (Rommelfanger et al., 2009; Mitrano et al., 2012; Velásquez-Martinez et al., 2012; Velásquez-Martínez et al., 2015) with alpha_1_-adrenergic receptors (α_1_-ARs) in the VTA serving as putative regulators of phasic DA efflux in the nucleus accumbens (NAc) (Goertz et al., 2015; Park et al., 2017). In our previous studies, we have shown that the mesolimbic VTA-NAc pathway, in contrast to the mesocortical (VTA–prefrontal cortex) pathway, is especially susceptible to modulation *via* intra-VTA α_1_-AR antagonists (Kielbinski et al., 2019). We have also shown that α_1_-AR microinfusion into the VTA potently inhibits cocaine seeking under extinction conditions, while sparing natural reward processing, i.e., having no effect on food seeking (Solecki et al., 2018, 2019a). Strikingly, we have previously found that the same attenuating effect of intra-VTA inhibition of α_1_-ARs is present for conditioned fear memories (Solecki et al., 2017). These observations suggest that NA in the VTA preferentially acts by modulating the response to highly salient environmental cues, irrespective of the hedonic value of such cues. However, to date, there have been no reports of the role of VTA α_1_-AR signaling in reward-based associative learning.

Here, we sought to further test this assumption. First, we compared the effects of intra-VTA microinfusion of the α_1_-AR antagonist prazosin in Pavlovian learning paradigms involving reinforcers with positive hedonic valance i.e., cocaine. In line with the idea that NA signaling is important for encoding salience, we found that in cocaine-induced conditioned place preference (CPP) paradigm antagonizing α_1_-ARs in the VTA leads to disruption of Pavlovian associative learning. Next, we asked whether the behavioral markers of positive hedonic valance, such as ability of cocaine to reinforce instrumental responding or evoke appetitive ultrasonic vocalizations (USVs) are affected by α_1_-AR inhibition–in both cases finding no effects of prazosin in doses otherwise effective in reducing cocaine-conditioned behaviors. Finally, we sought to confirm the proposed mechanism of prazosin action by assessing α_1_-AR distribution in the anterior part of the VTA targeted by our previous pharmacological manipulation of conditioned behaviors (Solecki et al., 2017, 2018, 2019a), as well as verify that the prazosin dose effective in shaping acquisition of memory in the CPP paradigm is indeed effective at reducing electrically induced phasic DA transients in NAc core. Taken together, these results solidify the concept of NA acting *via* α_1_-ARs in the VTA to modulate conditioned behavior by affecting the behavioral salience of environmental stimuli tied to positive outcomes.

## Materials and methods

### Animals

Male Sprague Dawley rats (280–350 g) were acquired from the Mossakowski Medical Research Centre PAS (Warsaw, Poland) and an Institute of Pharmacology PAS (Kraków, Poland) breeding facility. In addition, for immunostaining, male tyrosine hydroxylase (TH) IRES-Cre^+/–^ transgenic rats [SD-Th-cre^TM 1sage^ (Brown et al., 2013)] were bred in the Institute of Zoology and Biomedical Research (Jagiellonian University) breeding facility (Kraków, Poland) under a breeding license with Horizon Discovery (Vienna, Austria). Rats were housed five per cage until the surgical procedures, after which all animals were singly housed. Housing conditions constituted of a temperature- and humidity-controlled room (20–22°C, 40–50% humidity) and a 12-h light/dark cycle (lights on at 7 am, all procedures during light phase). All rats had *ad libitum* access to food and water. All experimental procedures were conducted according to the EU Guide for the Care and Use of Laboratory Animals and were approved by the Committee on the Ethics of Animal Experiments at the Institute of Pharmacology, Polish Academy of Sciences (Kraków, Poland) and the Committee on the Ethics of Animal Experiments at the Jagiellonian University.

### Drugs

Prazosin hydrochloride (Praz; 0.1–1 μg, Sigma-Aldrich, Poznań, Poland), a selective α_1_-AR antagonist, was dissolved in PBS and sonicated before microinjections. Prazosin was infused into the VTA in a 0.5 μL volume (Praz; 0.24–2.38 nmol/side) at a rate of 0.5 μL/min through the internal cannula using a Hamilton 25-gauge syringe, with additional 1 min during which the internal cannula was left in place to allow absorption of the drug. The drug doses were chosen based on previous work from our laboratory (Solecki et al., 2017, 2018) and others’ demonstrating the ability of prazosin administration to modulate behavior (Selken and Nichols, 2007; Do-Monte et al., 2010). Cocaine hydrochloride (COC) (15–25 mg/kg i.p., Sigma-Aldrich, Poznań, Poland) was dissolved in saline. The final injected volume was always 0.1 ml per 100 g of animal weight. The doses of COC for experiments studying the behavioral effects of the α_1_-AR antagonist were calculated based on initial dose-response experiments reported here.

### Intra-ventral tegmental area cannula implantation

Subjects underwent habituation to handling for 5 days prior to surgery. Rats were anesthetized with ketamine HCl (100 mg/kg, i.m., Biowet-Puławy, Poławy, Poland) and xylazine (10 mg/kg, i.m., Biowet-Puławy, Poland) and placed in a stereotaxic frame (Stoelting Europe, Dublin, Ireland) for intracranial cannula implantation. Bilateral guide cannulae with matching dummy cannulae and a dust cap (Plastics One, Roanoke, VA, United State) were placed dorsal to the VTA (AP −5.2 mm, ML ±0.5 mm, DV −7.0 mm). To ensure the stability of the implanted cannula, four screws (Agnthos, Lidingo, Sweden) and dental cement (Duracryl, SpofaDental, Jicia, Czechia) were positioned on the skull. After the surgery, all animals underwent post-operative care, as described previously. Rats were given at least a week to recover after the intra-VTA cannula implantation, with *ad libitum* access to food and water.

### Conditioned place preference

Conditioned place preference testing was conducted in a custom-made apparatus (black Plexiglas 80 × 80-cm arena with 60-cm-high black walls). The apparatus consisted of two conditioning chambers (27 × 27 × 40 cm) separated by guillotine doors and a central platform (15 × 7 × 40 cm). One of the conditioning chambers had white Plexiglas walls with vertical black stripes and a black Plexiglas floor with round recesses. The second chamber had white Plexiglas walls with horizontal black stripes and a rough black floor. The distinct visual and textural cues served as conditioned stimuli. The central platform differed from both conditioning chambers (gray and white Plexiglas walls and a smooth gray floor). Both chambers and the central platform were dimly illuminated with white light (10 lux in the middle of each chamber). 24 h prior to testing, all rats were transferred into the testing room for 4 h acclimatization.

**FIGURE 1:**
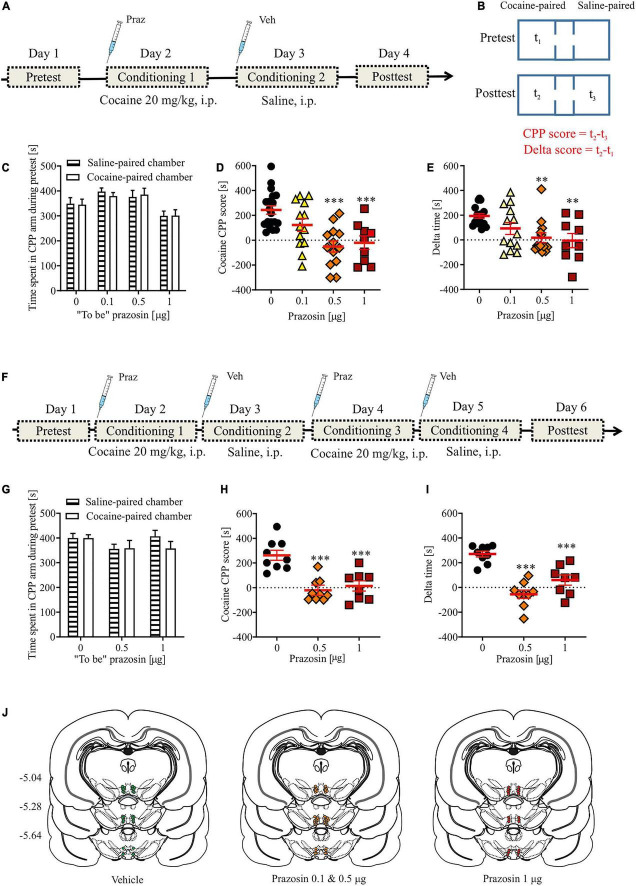
Effects of α_1_-AR blockade in the VTA on the acquisition of cocaine-evoked CPP. **(A,F)** The experimental time-line and schedule of intra-VTA microinfusions during the acquisition of CPP induced by one **(A)** or two **(F)** cocaine (20 mg/kg, i.p.) conditionings. **(B)** Graphical representation of the two methods of calculating conditioned responses in the CPP paradigm. CPP score is defined as a difference between time spent (t_2_) in the cocaine-paired chamber minus time spent in (t_3_) control chamber (saline-paired) during post-conditioning (post-test). Delta score is defined as a difference between time spent (t_2_) in the cocaine-paired chamber during post-conditioning minus time spent (t_1_) in the same chamber but during pre-conditioning (pre-test). **(C,G)** There were no pre-existing differences in the time spent in the cocaine- and saline-paired chambers during pre-test between future vehicle (Veh)- and prazosin (Praz)-treated subjects. **(D,E)** Intra-VTA microinfusion of prazosin attenuated the acquisition of CPP induced by one (**D** for CPP score and **E** for delta score) and two (**H** for CPP score and **I** for delta score) cocaine (20 mg/kg, i.p.) conditionings. **(J)** Localization of histologically verified cannula placements. Data are presented as individual data points as well as the mean and SEM. ^**^p < 0.01, ^***^p < 0.001, Newman–Keuls *post-hoc* test vs. Praz 0 μg.

The testing procedure consisted of three stages: a pre-conditioning test (day 1), conditioning (days 2–3 or 2–5), and a post-conditioning test (day 4 or 6). This CPP study design allowed the minimization of tissue damage associated with repeated transcranial microinfusions and a reliable measurement of the impact of intra-VTA manipulations on the tested behavior. Importantly, single-session drug conditioning in the CPP paradigm has been previously demonstrated (Bardo et al., 2003). The design of the experiment measuring intra-VTA α_1_-AR blockade effects on cocaine-induced CPP is shown in Figure 1A. In addition, to further demonstrate intra-VTA α_1_-AR blockade effects on reward-based Pavlovian associative learning, we performed a CPP experiment with two cocaine and two saline conditioning sessions. The design of this experiment is shown in Figure 1F. The design of the control experiment measuring intra-VTA α_1_-AR blockade effects on CPP is shown in Supplementary Figure 2A. Pre- and post-conditioning tests (15 min each), during which rats had access to all chambers, were recorded by a video camera, and time spent in each chamber was measured using ANY-maze software (Stoelting Europe, Ireland). After the pre-conditioning test, one chamber was paired with drug administration (conditioned chamber) and the other with vehicle (control chamber). Animals that displayed initial preference for one of the chambers (>200 s spent in one compartment in comparison to the other) were excluded from conditioning (*n* = 5). The assignment of treatment conditions to the chambers was counterbalanced (each chamber served as drug-paired and vehicle-paired with equal frequency). During conditioning, the rats were randomly assigned to treatment groups and were treated with saline or drug immediately before being placed in the appropriate chamber for 40 min. Intra-VTA microinfusion was performed immediately prior to each cocaine or saline administration. In addition, in case of CPP experiment with two sessions of cocaine conditioning (Figure 1F) cocaine and saline treatments were counterbalanced as half of the rats received prazosin (or vehicle) microinfusion prior to cocaine conditioning on day 2 and 4 and vehicle microinfusion prior to saline conditioning on day 3 and 5, whereas second half received vehicle microinfusion prior to saline conditioning on day 2 and 4 and prazosin (or vehicle) microinfusion prior to cocaine conditioning on day 3 and 5, followed by a post-conditioning test on day 6.

The apparatus was cleaned after each rat. We used two measures of place preference–the CPP score (Figure 1B; CPP score = time spend in the drug-paired–time spend in the vehicle-paired chamber during the post-conditioning testing) and delta score (Figure 1B; delta score = time spend in the drug-paired chamber during pre-conditioning–time spend in the drug-paired chamber during post-conditioning). The control group in the cocaine dose-response experiment received saline conditioning on day 2 instead of cocaine. The control group in the experiment testing prazosin effects on cocaine-induced CPP received intra-VTA vehicle (PBS) prior to cocaine conditioning (Figure 1). The control group in the intra-VTA prazosin dose-response experiment (Supplementary Figures 2A, B) received intra-VTA vehicle (PBS) administration on day 2 (conditioning 1) instead of prazosin.

### Cocaine self-administration and fixed ratio 1 testing

**FIGURE 2:**
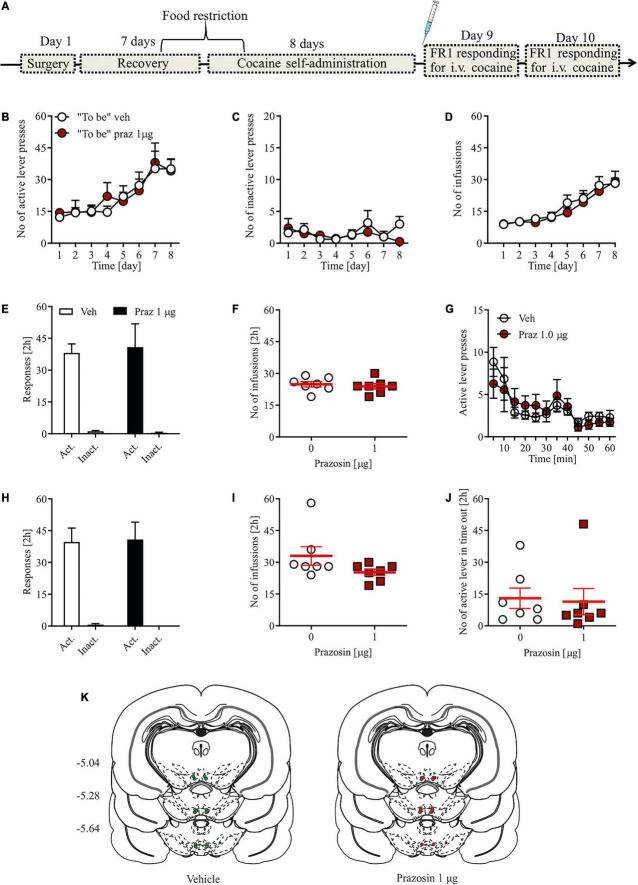
Effects of α_1_-AR blockade in the VTA on cocaine reinforcement in the cocaine self-administration paradigm. **(A)** Rats, after intravenous catheter and intra-VTA cannula implantation surgeries and recovery were trained to self-administer cocaine (∼0.5 mg/kg/inf.) for 8 days. On day 9, intra-VTA vehicle (Veh) or prazosin (Praz) microinfusions were performed immediately prior to subsequent cocaine self-administration session. **(B–D)** There were no pre-existing differences during cocaine self-administration training in the number of **(B)** active and **(C)** inactive lever presses as well as in **(D)** the number of cocaine infusions between future vehicle (Veh)- and prazosin (Praz)-treated subjects. **(E)** Intra-VTA microinfusion of prazosin (Praz 1 μg/side) on day 9 had no effect on the numbers of active and inactive lever responses and on **(F)** the number of cocaine infusions. **(G)** Intra-VTA α_1_-AR blockade had no effects on the numbers of active lever presses over time. Similarly, prazosin treatment on day 9 had no effects on **(H)** the numbers of active and inactive lever presses and **(I)** cocaine infusions, 24 h after treatment (on day 10). **(J)** Finally, prazosin microinfusion on day 9 had no effects on active lever responding during time-out. **(K)** Localization of histologically verified cannula placements. Data are presented as individual data points as well as the mean and SEM. Act, active lever; Inact, inactive lever.

The design of the experiment is shown in Figure 2A. Rats were anesthetized with ketamine HCl (100 mg/kg, i.p.) and xylazine (10 mg/kg, i.p.) and implanted with a catheter into the external jugular vein, as described previously (McFarland and Kalivas, 2001). Following the implantation, bilateral cannulae (Plastics One, Roanoke, VA, United States) were placed above the VTA, as described above.

Cocaine self-administration training started 7 days after surgery and was preceded by 2–3 days of food restriction to ∼90% of rats free-feeding body weight. Training constituted of 2 h daily sessions for eight consecutive days. During training session, each rat was placed in a standard operant chamber illuminated by a house light and equipped by two protruding levers, two stimulus lights and a tone generator (Med Associates, St. Albans, VT, United States). Rats were trained under a fixed ratio 1 (FR1) schedule of reinforcement during which each active lever depression led to an intravenous cocaine infusion (0.18 mg over 6 s, ∼0.5 mg/kg) and a conditioned stimulus (CS) cue presentation (tone + stimulus light for 6 s), followed by a 20 s time-out. Inactive lever depressions had no programed consequences. Majority of rats acquired stable (less than 15% variability in operant responding over last three consecutive days) self-administration behavior and those that did not (*n* = 4) were excluded from the study and were not tested for FR1 responding. After 8 days of cocaine self-administration and immediately prior to the self-administration training session on day 9, the rats were treated with an intra-VTA microinfusion (prazosin or vehicle) and placed in the operant chambers for 2 h of testing with the FR1 schedule of reinforcement described above. Next, on day 10, all rats received a final cocaine self-administration session with no intra-VTA microinfusions to control for any potential effects of prazosin administered the day before.

### Cocaine-evoked ultrasonic vocalizations recording, analysis, and classification

Cocaine (10 and 20 mg/kg, i.p.) or saline (i.p.) was administered immediately prior to rats being placed in the home cage for 30 min. Rat USVs were recorded with an UltraSoundGate Condenser Microphone CM16 (Avisoft Bioacoustics, Glienicke/Nordbahn, Germany) connected to an ultrasound-recording device (UltraSoundGate 116 Hb, Avisoft, Berlin, Germany) and to Avisoft RECORDER software (sampling at 200,000 Hz and 16-bit recording). Next, the recordings were transferred to Raven Pro 1.4 interactive sound analysis software (Cornell Lab of Ornithology, Bioacoustics Research Program, United States), and a fast Fourier transform (512 FFT-length, Hamming windows and 75% overlap) along with background noise filter (cut-off frequency of 20 kHz) were applied. Each call was selected manually by a trained investigator by using Raven Pro 1.4 software. Additionally, we investigated off-line the structure of the calls. Each identified 50-kHz call was classified into 1 of 14 distinct categories described previously (Wright et al., 2010).

### Locomotor activity

A separate cohort of rats was tested for locomotor activity after intra-VTA prazosin microinfusion. Immediately after drug microinfusion, rats were placed in the center of an open field apparatus (black Plexiglas 80 × 80-cm arena with 60-cm-high black Plexiglas walls illuminated with a light intensity of 20 lux) and left inside for 30 min. Locomotor activity was tracked by a video camera and analyzed using ANY-maze software (Stoelting Europe, Dublin, Ireland). The apparatus was cleaned using 70% ethanol and dried after each rat.

### Histological verification of cannula placement

To visualize the site of intra-VTA microinfusions, 2% solution of Chicago Sky-Blue dye (Sigma-Aldrich, Poznań, Poland) in PBS was bilaterally microinjected into the VTA (0.5 μL/1 min) in anesthetized (pentobarbital 150 mg/kg i.p., Biowet-Puławy, Puławy, Poland) subjects. Immediately after microinfusion, rats were decapitated, and their brains were removed and placed in a 4% solution of paraformaldehyde for 72 h. Next, brains were sliced (200 μm) with a vibratome (VT1000S, Leica Biosystems, Wetzlar, Germany), and the diffusion of the dye was analyzed. All data from subjects with misplaced cannulae were removed from the analysis of intra-VTA α_1_-AR antagonist effects (Table 1). All cannula placements are shown in Supplementary Figure 1.

**TABLE 1:** Number of subjects with the VTA cannula hits and misplacements.

Behavioral test	Drug treatment	VTA hits	VTA misplacements
CPP	Veh	12	n.a.
	Praz 0.5	14	n.a.
	Praz 1	10	n.a.
OF	Veh	8	n.a.
	Praz 0.5	7	1
	Praz 1	7	1
Cocaine-induced CPP (one conditioning)	Veh	18	n.a.
	Praz 0.1	13	4
	Praz 0.5	13	4
	Praz 1	9	3
Cocaine-induced CPP (two conditioning)	Veh	9	1
	Praz 0.5	9	n.a.
	Praz 1	8	3
FR1 responding for i.v. cocaine	Veh	7	1
	Praz	7	1
Saline-evoked USVs	Veh	14	n.a.
	Praz	8	n.a.
Cocaine (10 mg/kg)-evoked USVs	Veh	7	1
	Praz	7	1
Cocaine (20 mg/kg)-evoked USVs	Veh	18	4
	Praz	23	6
FSCV	Praz 1	7	n.a.
Total		235	31 (13.2%)

CPP, conditioned place preference; OF, open field test; FR1, fixed ratio 1; USVs, ultrasonic vocalizations; Veh, PBS; Praz, prazosin; n.a., not applicable. Prazosin doses in μg/side.

### Immunohistochemistry

IRES-Cre^+/–^ rats underwent virus transduction (AAV5-EF1a-DIO-EYFP; 4.4 × 10^12^ virus molecules/ml; University of North Carolina Viral Core) for phototagging of the TH-positive neurons. First, rats were anesthetized with ketamine HCl (100 mg/kg, i.m., Biowet-Puławy, Puławy, Poland) and xylazine (10 mg/kg, i.m., Biowet-Puławy, Puławy, Poland), placed in a stereotaxic frame (Stoelting Europe, Dublin, Ireland) and a sagittal skin incision at the top of the head and a small hole in the skull above the VTA was made. Next, AAV was administered (0.1 μl/1 min; 0.5 μl) into the VTA as described before (Solecki et al., 2019a,b). The stereotaxic coordinates used for microinjection were referenced from Bregma: AP −5.2 to −5.3 mm, ML ±0.7 mm, and DV −8.0 mm (Paxinos and Watson, 2013). Finally, the incision above the skull was sutured using silk sutures. After the surgery, animals underwent post-operative care as previously described (Solecki et al., 2019a) and were given 4 weeks to recover after the procedure and to ensure effective virus transduction (Witten et al., 2011).

For immunostainings, rats were anesthetized with pentobarbital (150 mg/kg i.p., Biowet-Puławy, Puławy, Poland) and perfused transcardially with 0.9% NaCl followed by 4% formalin (Chempur, Piekary Śla̧skie, Poland). Immediately after the perfusion, animals were decapitated; their brains were removed and post-fixed in PBS containing 4% formalin at 4°C. Next, brains were sliced with a vibratome (model VT1000S, Leica Biosystems, Wetzlar, Germany) to coronal brain sections (40 μm). For each animal, 6–12 sections were acquired at the level of the VTA. Slices were placed in 0.01% sodium azide solution (K305.1; Roth, Germany) for immunohistological procedures. For S-100B protein, glutamic acid decarboxylase (GAD), α_1_-AR staining tissue sections were immersed in a blocking solution consisting of 5% normal goat serum (G9023; Sigma-Aldrich, Poznań, Poland) dissolved in PBST (0.5% Triton X-100 with 0.1% PBS). For GABAergic neurons, anti-GAD67 antibody (1:5000, MAB 5406, Merck Millipore, Burlington, MA, United States) was used. Anti-S-100B protein antibody (1:1000, S-2532, Sigma-Aldrich, Poznań, Poland) was used to identify glial cells. GAD67 and S-100B-positive cells were detected using a goat anti-mouse Alexa Fluor 555 (1:500, A21422, Life Technologies, Warsaw, Poland) secondary antibody. The expression of α1-AR was confirmed by labeling with rabbit anti-alpha1-adrenoreceptor antibody explorer kits (1:250, AK-490, Alomone Labs, Jerusalem, Israel) and detected with a goat anti-rabbit Alexa Fluor 568 (1:500, A11011, Life Technologies, Warsaw, Poland) and goat anti-rabbit Alexa Fluor 488 (1:500, A11034, Life Technologies, Warsaw, Poland) secondary antibody. Cell nuclei were identified using the 4′,6-diamidino-2-phenylindole dihydrochloride (1 μg/1 ml, D9542, Sigma-Aldrich, Poznań, Poland). The specificity of anti-α_1_-AR antibody was tested by pre-incubation with manufacturer-supplied control antigen peptides (1:125, AK-490, Alomone Labs, Jerusalem, Israel) and comparing the strength of the signal between the control and blocked antibodies. The immunostaining was evaluated by confocal microscopy (LSM 710 on Axio Observer Z1 microscope; Zeiss, EC Plan-Neofluar 10×/0.30 M27 objective) and ImageJ software (Schneider et al., 2012) by imaging the EYFP and α_1_-AR immunoreactive cells.

### Fast-scan cyclic voltammetry

Effect of intra-VTA α_1_-AR blockade on electrically evoked phasic DA release in the forebrain was performed as previously described for intra-VTA pharmacological manipulations (Solecki et al., 2013; Kielbinski et al., 2019). We used within subject experiment design as previously described (Wickham et al., 2015; Kielbinski et al., 2019). Rats were anesthetized (urethane 1.5 g/kg, ip.) and placed in a stereotaxic frame to lower an electrode (bipolar, stainless-steel stimulating electrode, Plastics One, Roanoke, VA, United States) into the VTA (AP −5.2 mm, ML −1.0 mm, and DV between −7.6 and −8.5 mm). Next, a carbon fiber microelectrode [described in Kielbinski et al. (2019)] was lowered into the NAc core (AP +1.2 mm, ML −1.4 mm, and DV from −6.2 to −6.9 mm) and a reference electrode (Ag/AgCl) was placed in contralateral hemisphere. To induce redox reactions, a triangular waveform (−0.4 to +1.3 V and back to −0.4 V at a rate of 400 V/s, repeated at 100 ms intervals) was applied to the microelectrode. To evoke phasic DA release, electrical stimulation consisted of biphasic square wave pulses (300 μA, 24 pulses at 60 Hz with 2 ms/pulse) were delivered to the VTA using stimulator (NL 800A, Neurolog, Digitimer Ltd., Hertfordshire, United Kingdom combined with PCI card, National Instruments, Austin, TX, United States). Electrical stimulation consisted of biphasic square wave pulses (300 μA, 24 pulses at 60 Hz with 2 ms/pulse) applied every 3 min. Obtained data was digitized and processed using NI-6711 and NI-6251 DAC/ADC cards (National Instruments, Austin, TX, United States) and High Definition Cyclic Voltammetry analysis software (University of North Carolina at Chapel Hill, NC, United States). Background-subtracted cyclic voltammograms (CVs) were obtained by digitally subtracting stable background currents to resolve CVs for dopamine.

To study the effects of intra-VTA prazosin administration, we used within subject experiment design as previously described (Wickham et al., 2015; Kielbinski et al., 2019). Briefly, once stimulated DA was detected in the NAc core, minimum of six baseline dopamine samples were collected, followed by six samples after the intra-VTA PBS administration, additional six traces for second baseline prior to prazosin administration and final 6–12 traces after prazosin microinfusion. All dopamine samples were collected at 3 min intervals. All infusions were made at the rate of 0.5 μL/min in the volume of 0.5 μL (1 min total infusion time) and the internal cannula remained in the guide cannula for 1 min to allow absorption. PBS data was normalized to the first baseline period, while the prazosin data was normalized to a second baseline.

### Data analysis

Data were analyzed for normal distribution using the Kolmogorov–Smirnov test (Statistica 13.3, Stat-Soft, Poland or GraphPad Software, San Diego, CA, United States). Behavioral effects of systemic cocaine or intra-VTA prazosin administration in the CPP and the open field tests were analyzed using Student’s *t*-test or one-way ANOVA (GraphPad Software, San Diego, CA, United States). The effects of intra-VTA prazosin administration on lever presses during the 2 h cocaine-taking test were analyzed using two-way ANOVA with prazosin dose and lever as factors (GraphPad Software, San Diego, CA, United States). Similarly, the effects of intra-VTA prazosin administration on the number of cocaine evoked USV and the USVs categories were analyzed using two-way ANOVA with prazosin dose and cocaine dose or prazosin dose and USV category as factors. For analysis of lever responding during cocaine self-administration training over days, a three-way repeated-measures ANOVA was conducted. Similarly, for analysis of number of infusions during cocaine self-administration training, active lever responding during FR1 test or stimulated phasic DA release over time, a two-way repeated-measures ANOVA was performed. If there was a significant main effect or a significant interaction, a subsequent Newman–Keuls *post hoc* analysis was performed. Supplementary Table 1 presents the factors and levels of ANOVA according to the drug microinfusions and the performed behavioral tests. Statistical significance was set at *p* < 0.05. All data values are presented as the means and SEM.

## Results

### Intra-ventral tegmental area administration of alpha_1_-adrenergic receptor antagonist attenuates acquisition of cocaine-induced place preference

To study the effect of single intra-VTA infusions of prazosin on the acquisition of cocaine-induced CPP, we first established a cocaine-induced CPP paradigm with a single cocaine conditioning session. Our results indicated that 20 mg/kg of cocaine is sufficient to induce cocaine CPP after only one cocaine conditioning session (Table 2); therefore, that dose was chosen for subsequent studies.

**TABLE 2:** Systemic (i.p.) administration of cocaine (20 mg/kg but not 15 or 25 mg/kg) supported the acquisition of cocaine-induced CPP after only one cocaine-chamber pairing.

Cocaine-induced CPP	*n*	CPP score (mean ± SEM)	ANOVA	*Post-hoc* test (vs. sal)	Delta score (mean ± SEM)	ANOVA	*Post-hoc* test (vs. sal)
Saline	6	−37 ± 119.3	*F*_(3,23)_ = 4.05, *p* < 0.05	n.a.	1.66 ± 66.7	*F*_(3,23)_ = 2.33, *p* < 0.05	n.a.
Coc 15 mg/kg	6	146.5 ± 61.5		n.s.	121.5 ± 58.1		n.s.
Coc 20 mg/kg	7	233.1 ± 80.2		*	237.2 ± 153.4		*
Coc 25 mg/kg	6	−91.2 ± 104.3		n.s.	14.8 ± 64.1		n.s.

Coc, Cocaine; n.a., not applicable; n.s., not significant. Data are presented as the mean ± SEM. *p < 0.05, Newman–Keuls post-hoc test vs. saline treated group.

Intra-VTA microinfusion of 0.5 or 1 μg of prazosin prevented the acquisition of cocaine-induced place preference (Figure 1D; *F*_(3,__50)_ = 10.19, *p* < 0.001; *post-hoc* test *p* < 0.001 vs. control). These findings were confirmed by an alternative analysis of the CPP results which allows the consideration of changes in behavior over days (Figure 1E; delta time analysis: *F*_(3,__50)_ = 6.01, *p* < 0.01, *post-hoc* test *p* < 0.01 for Praz 0.5 μg and Praz 1 μg vs. control; time spent in cocaine-paired arm during pre- and post-test shown in Supplementary Figure 2A). These effects were not due to pre-existing differences in the initial time spent in the two CPP chambers by future vehicle- and prazosin-treated rats, as those groups did not differ in the time spent in the control vs. conditioned chambers during the pre-test session (Figure 1C; chamber: *F*_(1,__98)_ = 0.02, *p* = 0.87; treatment × chamber interaction: *F*_(2,__98)_ = 0.12, *p* = 0.94).

To further examine whether the effects of intra-VTA microinfusion of prazosin would be present after more than one cocaine conditioning pairing, we performed an additional experiment in which subjects received two saline-chamber and two cocaine-chamber pairings followed by a post-test session. Similar to previous results, intra-VTA microinfusion of prazosin subsequently prevented the acquisition of cocaine-induced CPP (CPP score: Figure 1H: *F*_(2,__23)_ = 17.43, *p* < 0.001, *post-hoc* test *p* < 0.001 for Praz 0.5 μg and Praz 1 μg vs. control; delta score: Figure 1I: *F*_(2,__23)_ = 25.97, *p* < 0.001, *post-hoc* test *p* < 0.001 for Praz 0.5 μg and Praz 1 μg vs. control; time spent in cocaine-paired arm during pre- and post-test shown in Supplementary Figure 2B). These effects were not due to pre-existing differences in the initial time spent in the two CPP chambers by future vehicle- and prazosin-treated rats (Figure 1G; chamber: *F*_(1,__46)_ = 0.57, *p* = 0.45; treatment × chamber interaction: *F*_(2,__46)_ = 0.8, *p* = 0.45).

To exclude potential dysphoric effects of intra-VTA prazosin treatment, we performed CPP in which one conditioning compartment was paired with intra-VTA prazosin and the other with vehicle (PBS) microinfusion (the experiment timeline is shown in Supplementary Figure 3A). Single prazosin conditioning did not support acquisition of place aversion nor preference (CPP score: Supplementary Figure 3C; *F*_(2,__33)_ = 0.03, *p* = 0.97; delta score: Supplementary Figure 3D; *F*_(2,__33)_ = 0.82, *p* = 0.44). These results were not due to pre-existing differences in the time spent in the two conditioning chambers in future vehicle- and prazosin-treated rats, as these groups showed no differences in time spent in the control vs. conditioned chambers during the pre-test (Supplementary Figure 3B; chamber: *F*_(1,__66)_ = 0.64, *p* = 0.8; treatment × chamber interaction: *F*_(2,__66)_ = 1.98, *p* = 0.14).

Intra-VTA α_1_-AR blockade might affect locomotion, as decreasing VTA activity transiently attenuates locomotor activity (Solecki et al., 2013), which could alter behavioral responses to the US as well as impair associative learning (Beninger, 1983). Prazosin treatment (0.5 and 1 μg) had no effects on total distance traveled during 30 min in the open field (Supplementary Figure 3E; *F*_(2,__19)_ = 0.38, *p* = 0.68). Similarly, closer examination of locomotion in 5-min time epochs revealed no effects of prazosin (Supplementary Figure 3F; treatment × time interaction: *F*_(10,__175)_ = 1.11, *p* = 0.71; treatment: *F*_(2,__175)_ = 0.81, *p* = 0.76). In addition, prazosin did not block habituation to the open field, as locomotor activity decreased over time similarly in both prazosin and control groups (Supplementary Figure 3F; time: *F*_(5,__175)_ = 113.31, *p* < 0.001; *post-hoc* test *p* < 0.01 vs. 5 min).

Together, our results demonstrating that intra-VTA prazosin administration potently and selectively attenuates acquisition of cocaine-induced CPP suggest that α_1_-AR blockade impaired associative learning, as previously suggested for the acquisition of fear memories (Solecki et al., 2017). Alternatively, α_1_-AR blockade might modulate rats’ sensitivity to the rewarding properties of cocaine, as intra-VTA prazosin administration attenuates cocaine-evoked phasic DA release in the NAc shell (Goertz et al., 2015).

To address the later alternative, we studied the effects of intra-VTA prazosin microinfusion in separate tests measuring cocaine behavioral effects (FR1 responses in a cocaine self-administration paradigm and in the number of cocaine-evoked positive affective USVs).

### Intra-ventral tegmental area administration of alpha_1_-adrenergic receptor antagonist did not change the number of fixed ratio 1 responses in a cocaine self-administration procedure

To test whether intra-VTA α_1_-AR blockade is sufficient to modulate the reinforcing properties of cocaine, we chose a single effective dose of the selective α_1_-AR antagonist prazosin that disrupted acquisition of cocaine-induced CPP. Prazosin (1 μg) microinfusion had no effects on instrumental responding for cocaine as shown by the similar numbers of active and inactive lever presses in prazosin- and vehicle-treated animals (Figure 2E; dose × lever interaction: *F*_(1,__24)_ = 0.06, *p* = 0.76; treatment: *F*_(1,__24)_ = 0.01, *p* = 0.85), and the differentiation between the active and inactive levers (lever: *F*_(1,__24)_ = 54.84, *p* < 0.0001 followed by *post-hoc* test *p* < 0.0001). In addition, prazosin treatment had no effect on the number of cocaine infusions earned during a 2-h cocaine self-administration session (Figure 2F; Student’s *t*-test: *t* = 0.1, df = 12, *p* = 0.91) or number of active lever presses during time-out (Figure 2J; Student’s *t*-test: *t* = 0.34, *df* = 12, *p* = 0.74). Further examination of 5-min epochs during the 2-h session confirmed that there were no transient effects of prazosin treatment (Figure 2G; treatment × time interaction: *F*_(11,__143)_ = 1.58, *p* = 0.68; treatment: *F*_(1,__13)_ = 1.59, *p* = 0.94; time: *F*_(11,__143)_ = 6.16, *p* < 0.0001). The lack of prazosin effects was not masked by potential pre-existing differences during cocaine self-administration training because future control subjects did not differ in the number of instrumental responding (Figures 2B,C; treatment × day × lever interaction: *F*_(7,__196)_ = 0.21, *p* = 0.98; treatment × day interaction: *F*_(7,__196)_ = 0.34, *p* = 0.93; treatment: *F*_(1,__28)_ = 0.07, *p* = 0.93; day: *F*_(7,__98)_ = 8.6, *p* < 0.001; lever: *F*_(1,__28)_ = 138.22, *p* = 0.0001) and cocaine infusions (Figure 2D; treatment × day interaction: *F*_(7,__98)_ = 0.27, *p* = 0.96; treatment: *F*_(1,__14)_ = 0.26, *p* = 0.61; day: *F*_(7,__98)_ = 18.81, *p* < 0.001) from the future prazosin-treated animals. We also ran a subsequent FR1 session 24 h after the prazosin treatment to ensure that no prolonged effects of intra-VTA α_1_-AR blockade were present. Prazosin microinfusion had no effects 24 h later on the numbers of active and inactive lever presses (Figure 2H; dose × lever interaction: *F*_(1,__24)_ = 0.09, *p* = 0.65; treatment: *F*_(1,__24)_ = 0.03, *p* = 0.73) or on the number of cocaine infusions earned during a 2-h cocaine self-administration session (Figure 2I; Student’s *t*-test: *t* = 0.07, df = 12, *p* = 0.66). All cannula placements are shown in Figure 2K.

These results, showing similar rates of instrumental responses for cocaine infusion in well-trained subjects after intra-VTA prazosin, suggest that the VTA α_1_-AR blockade did not alter sensitivity to the reinforcing properties of cocaine in the self-administration paradigm or hinder the subjects’ ability to perform the task itself.

### Alpha_1_-adrenergic receptor blockade in the ventral tegmental area did not affect the number of cocaine-evoked appetitive ultrasonic vocalizations

**FIGURE 3:**
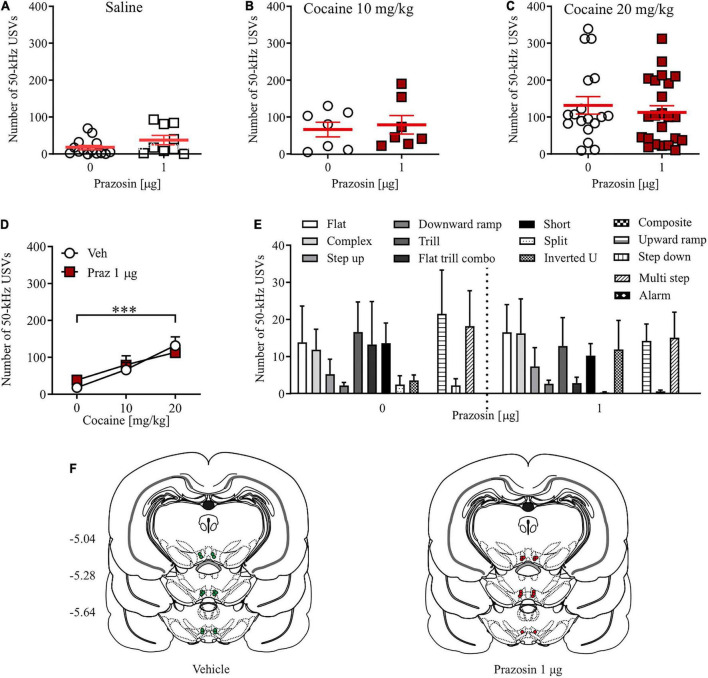
Effects of α_1_-AR blockade in the VTA on cocaine-evoked 50-kHz USVs. α_1_-AR blockade in the VTA with 1 μg/side prazosin (Praz) had no effect on the number of appetitive USVs after **(A)** saline (i.p.) or cocaine administration at **(B)** 10 mg/kg, i.p. or **(C)** 20 mg/kg dose. **(D)** Intra-VTA prazosin infusion had no effect on the cocaine-induced increase in the number of appetitive USVs and **(E)** did not modulate selected categories USVs after cocaine (20 mg/kg, i.p.) administration. **(F)** Localization of histologically verified cannula placements. Data are presented as individual data points as well as the mean and SEM. ^***^p < 0.001, Newman–Keuls *post-hoc* test vs. Cocaine 0 mg/kg.

Prazosin treatment had no effects on the number of 50-kHz USVs emitted by cocaine naïve subjects (Figure 3A; Student’s *t*-test: *t* = 1.63, df = 21, *p* = 0.13). Similarly, intra-VTA administration of 1 μg prazosin did not alter the number of appetitive USVs evoked by 10 mg/kg (Figure 3B; Student’s *t*-test: *t* = 0.73, df = 13, *p* = 0.69) or 20 mg/kg cocaine (Figure 3C; Student’s *t*-test: *t* = 0.16, df = 42, *p* = 0.87). This indicates that the positive affect associated with systemic cocaine administration at a dose supporting reward-based Pavlovian associative learning in the CPP paradigm (20 mg/kg) is not modulated by the VTA α_1_-AR signaling (Figure 3D; cocaine dose × prazosin treatment interaction: *F*_(2,__72)_ = 0.58, *p* = 0.56; prazosin treatment: *F*_(1,__72)_ = 0.66, *p* = 0.79; cocaine dose: *F*_(2,__72)_ = 11.72, *p* < 0.001, *post-hoc* test *p* < 0.001). Closer examination of appetitive USVs supported those observations, as prazosin treatment did not change the number of any single category of cocaine-evoked appetitive USV calls (Figure 3E; dose × USV category interaction: *F*_(12,__546)_ = 0.33, *p* = 0.98; treatment: *F*_(1,__546)_ = 0.19, *p* = 0.65; USV category: *F*_(12,__546)_ = 0.33, *p* < 0.01). All cannula placements are shown in Figure 3F. Together, these results indicate that the positive affect-inducing properties of cocaine are not regulated by VTA α_1_-AR signaling.

### Localization of alpha_1_-adrenergic receptor expression in cells in the anterior part of the ventral tegmental area

**FIGURE 4:**
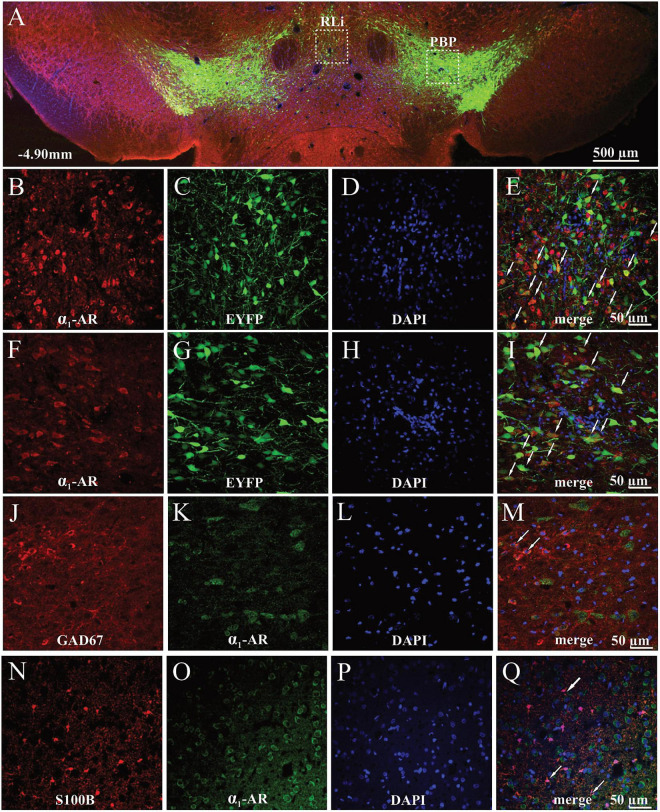
Localization of α_1_-AR-expressing cells in the anterior parts of the VTA of TH-cre^+^ rats. **(A)** Representative image of the EYFP- and α_1_-AR-expressing cells. **(B–E)** Magnified cells with α_1_-AR expression (red), EYFP (green), DAPI (blue), and merged channels showing α_1_-AR-immunoreactive and EYFP-expressing cells (white arrows) in the medial part of the anterior VTA. **(F–I)** Magnified cells with α_1_-AR (red), EYFP (green), DAPI (blue), and merged channels showing co-localized α_1_-AR-positive and EYFP-expressing cells (white arrows) in the lateral part of the anterior VTA. **(J–Q)** α_1_-AR expression in astrocytes (S100B) and GAD67-positive GABAergic interneurons. **(J–M)** Magnified images of astrocytes (red), α_1_-AR immunostaining (green), DAPI (blue), and composite images showing co-localization (white arrows) between α_1_-AR and S100B expression. **(N–Q)** Magnified images of GAD67-positive interneurons (red), α_1_-AR expression (green), DAPI (blue), and composite images showing co-localization between α_1_-AR and GAD67 immunoreactivity (white arrows). PBP, parabrachial pigmented nucleus; RLi, rostral linear nucleus.

To provide immunohistochemical evidence for the localization of α_1_-AR in the VTA we used a combined immunohistochemical and genetic approach which allowed us to differentiate DAergic neurons expressing EYFP from NAergic processes, as reported previously (Solecki et al., 2019b). Qualitative analysis of the α_1_-AR immunostaining demonstrated that α_1_-AR-positive cells were widely distributed in both the medial part of the anterior VTA corresponding to rostrolinear (RLi) and interfascicular nuclei (IF) (Figures 4A–E), as well as the lateral part of anterior VTA, such as the parabrachial pigmented nucleus (PBP) (Figures 4A,F–I). In those areas, α_1_-AR immunoreactivity was predominantly found in the cell bodies of TH-positive neurons tagged with EYFP. Importantly, combined immunohistochemical and genetic approach allowed us to differentiate DAergic neurons expressing EYFP under the control of the TH promoter from NAergic processes. Sparse co-localization between α_1_-AR and the GABAergic interneuron marker GAD67 staining (Figures 4J–M) as well as between α_1_-AR and the astrocytic marker S100B (Figures 4N–Q) was also observed. Likewise, α_1_-AR in those cells was present mainly in the cell bodies.

### Alpha_1_-adrenergic receptor blockade in the ventral tegmental area attenuates phasic dopamine release in the nucleus accumbens core

**FIGURE 5:**
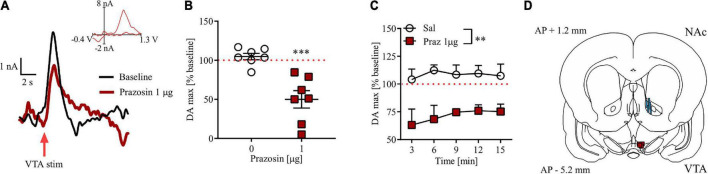
Effects of prazosin (Praz) on electrically evoked phasic dopamine release in the NAc core. **(A)** Representative FSCV traces obtained after intra-VTA infusion of saline (baseline) and 1.0 μg prazosin. Insets show representative current to voltage traces. **(B)** Maximal effects (shown as% of their respective baseline) of intra-VTA administration of prazosin on DA release in the NAc core shown as relative DA at the time point at which highest mean difference was observed. **(C)** Time course of relative DA peaks over five consecutive measurements after saline (Sal) and 1.0 μg Prazosin. **(D)** Localization of histologically verified cannula and FSCV electrode placements. ^**^p < 0.01, ^***^p < 0.001. Stimulation (stim). Data are shown as **(B)** individual data points and/or **(B,C)** mean ± SEM.

Intra-VTA prazosin microinfusion potently attenuated electrically evoked phasic DA release in the NAc core (Figure 5A). Prazosin maximal effect was displayed around 3 min after infusion and resulted in 49% of baseline DA release preceding intra-VTA α_1_-AR blockade (Figure 5B; *t* = 8.04, df = 6, *p* < 0.001). These effects were stable over 15 min in anesthetized subjects (Figure 5C; drug effect *F*_(1,__12)_ = 14.87, *p* < 0.01, *post-hoc* test *p* < 0.01; time *F*_(4,__48)_ = 0.33, *p* = 0.60; drug × time *F*_(4,__48)_ = 0.33, *p* = 0.85). All electrode and cannula placements are shown in Figure 5D.

## Discussion

Here, we studied the physiological and behavioral effects of intra-VTA α_1_-AR blockade using the selective α_1_-AR antagonist prazosin. Our data show that intra-VTA α_1_-AR blockade potently impairs Pavlovian association learning with positive outcome. Such regulatory action of α_1_-ARs requires their presence in the VTA, as well as the presence of afferents from which the noradrenaline is released. Finally, modulation of noradrenergic singling in the VTA should impact the physiology of the DA circuitry. Indeed, those crucial substrates for NAergic modulation are in place, as evidenced by the presence of α_1_-AR immunoreactivity in virally labeled DAergic neurons, presence of noradrenergic afferents as well as the observed inhibition of phasic DA outflow in the NAc by intra-VTA prazosin microinfusion.

### Physiological relevance of alpha_1_-adrenergic receptor in the ventral tegmental area

The NAergic system has well-established and dense projections into the VTA, with innervation arising primarily from the locus coeruleus (LC) as well as areas A1 and A2 (Mejías-Aponte et al., 2009; Robertson et al., 2013; Mejias-Aponte, 2016). NA release in the VTA is posited to be primarily performed *via* volume transmission from varicosities on NAergic neurons (Liprando et al., 2004), suggesting that any given NA release event can stimulate multiple cellular components adjacent to the release site. Expression of ARs in the VTA is also fairly well established, with initial experiments locating α_1_-ARs in the VTA with radioligand binding assays (Aston-Jones, 1985). These receptors are functional, with both LC stimulation and intra-VTA α_1_-AR agonist administration being capable of increasing VTA DA neuron firing (Grenhoff and Svensson, 1993; Grenhoff et al., 1993). Precise localization of α_1_-AR is challenging, however. Microstructural electron microscopy studies point to expression predominantly on neuronal terminals in the vicinity of both inhibitory (*symmetric*) and excitatory (*asymmetric*) synapses, with a contribution of other compartments, such as neuronal and glial cell bodies (Rommelfanger et al., 2009).

All of these cellular compartments serve as viable targets for neuromodulatory action of NA. This includes DA neurons themselves (Grenhoff and Svensson, 1993; Grenhoff et al., 1993; Goertz et al., 2015), non-DA cells, which are putative interneurons (Paladini and Williams, 2004; Pradel et al., 2018), as well as both GABAergic and glutamatergic terminals (Velásquez-Martinez et al., 2012; Velásquez-Martínez et al., 2015). In this study we show, using immunofluorescence, putative α_1_-AR expression on both DA and GABA neuronal cell bodies, as well as S100B-positive astrocytes. Notably, while it is unknown how astrocytes in the VTA respond to NA, in other brain structures these cells have been implicated in shaping the reciprocal potentiation of glutamatergic and NA signaling underlying the effects of arousal, making glial cells a potential target for future studies on their role in encoding salience by potentially increasing the glutamatergic drive onto DA neurons in behavioral situations where high NA tone and elevated activity at glutamatergic afferents coexist (Paukert et al., 2014; Perea et al., 2016; El et al., 2018; Kofuji and Araque, 2020; Oe et al., 2020).

In lieu of additional evidence of astrocytic involvement in potentiating DA release from the VTA in response to NA, DAergic neurons themselves constitute the most parsimonious potential target. Electrochemical evidence from fast-scan cyclic voltammetry (FSCV) experiments strongly suggests that α_1_-ARs in the VTA modulate DA release in target structures of the forebrain, especially NAc core and shell (Goertz et al., 2015; Park et al., 2017). Indeed, we have recently shown that this regulatory effect is heterogeneous, and confined mostly to NAc-projecting neurons forming the mesolimbic, as opposed to mesocortical, pathway, and limited to phasic DA release, with no effects of intra-VTA α_1_-AR inhibition on tonic DA release (Kielbinski et al., 2019). This observation potentially favors a direct, post-synaptic effect *via* I_*h*_/SK channels (Goertz et al., 2015), given the evidence that DA neurons constituting the mesolimbic pathway, but not DA neurons projecting to the mPFC, express high levels of those channels (Juarez and Han, 2016). Corroborating this hypothesis is the recent observation that behavioral sensitization to cocaine by repeated exposure also desensitized α_1_-AR located on glutamatergic, but not GABAergic terminals (Velasquez-Martinez et al., 2020). Yet, in previous studies, we have shown that α_1_-AR blockade in the VTA remains effective in attenuating cocaine seeking behavior despite the necessary prolonged pre-exposure to cocaine (Solecki et al., 2018, 2019a). Here, we support the hypothesis that phasic DA release in the NAc core is a potential downstream mechanism for α_1_-AR-mediated modulation of behavior, by showing that intra-VTA prazosin, at the dose effective at inhibiting Pavlovian learning (and previously shown to attenuate cocaine seeking), is also sufficient to decrease evoked phasic DA in NAc core. We propose that facilitation of phasic DA release in this circuit is a potential mechanism through which NA promotes salience encoding in the VTA.

### The role of alpha_1_-adrenergic receptor in the ventral tegmental area in Pavlovian association learning

Cocaine has similar affinity to the DA transporter (DAT) and NA transporter (NET) (Reith et al., 1997; Han and Gu, 2006). Indeed, the levels of both neurotransmitters are significantly elevated by stimulant drugs, including cocaine. Moreover, NA, acting *via* α_1_-ARs, contributes to hyperlocomotion and behavioral sensitization in response to cocaine (Schmidt and Weinshenker, 2014). How crucial is NA in mediating the rewarding effects of cocaine, and which aspects of cocaine-associated behavior depend on AR-mediated signaling, is not completely clear. While limited clinical evidence seems to suggest that systemic α_1_-AR antagonist administration is capable of attenuating positive subjective effects of cocaine (Newton et al., 2012), neither α_1_-AR agonists nor antagonists have been conclusively demonstrated to decrease the rewarding effects of cocaine in drug discrimination or drug substitution assays (Kleven and Koek, 1997, 1998; Ercil and France, 2003; Schmidt and Weinshenker, 2014). Our results, demonstrating no effects of intra-VTA prazosin on cocaine-reinforced operant responding in the cocaine self-administration paradigm and on cocaine-induced increases in positive affect (measured as appetitive USVs) indicate that intra-VTA α_1_-AR blockade does not change the reinforcing or positive affect-inducing effects of cocaine, despite its ability to attenuate the acquisition of cocaine-induced place preference.

The lack of effect of intra-VTA α_1_-AR blockade on cocaine reinforcement of instrumental response is in line with previously reported results (Ecke et al., 2012). Similarly, we have previously reported analogous findings in conditioned fear–a behavioral paradigm involving associative learning with aversive, rather than rewarding, stimuli (Solecki et al., 2017). In that study, intra-VTA α_1_-AR blockade did not affect anxiety-like behaviors measured in the open field or the light/dark box and had no effect on locomotor activity. In addition, intra-VTA α_1_-AR blockade during fear memory acquisition had no effects on the magnitude (measured as length in s) of dysphoric 22 kHz USVs, while at the same time attenuating stress responses such as freezing during fear memory recall (Solecki et al., 2017). Similar results across those two paradigms (fear conditioning, cocaine CPP) suggest that α_1_-AR signaling in the VTA is involved in all instances of Pavlovian association learning, regardless of outcome (positive or negative).

Indeed, taken together, our results lead us to a more generalized conclusion that intra-VTA α_1_-AR signaling does not regulate affective state. Accordingly, and in line with earlier studies which used systemic administration (Zarrindast et al., 2002; Sahraei et al., 2004), intra-VTA prazosin microinfusion was not sufficient to support the acquisition of Pavlovian conditioning measured in the CPP paradigm. Thus, while α_1_-AR signaling in the VTA is necessary for the acquisition of Pavlovian associations regardless of their hedonic valance, it does not affect the intrinsic rewarding or aversive properties of stimuli, nor does it exhibit sedative, dysphoric or reinforcing effects on its own. Instead, we hypothesize that salient environmental challenges (such as exposition to electric shock or cocaine) increase NA release in the VTA, and *via* α_1_-AR, enhance burst firing of DAergic neurons (Goertz et al., 2015), enabling salience detection and subsequent attribution of salience and valence to the CSs. In such a context, intra-VTA NA signaling would have a permissive role in the CS-US associations regardless of US hedonic valance, due to its role in salience encoding. Importantly, this hypothesized mechanism of α_1_-AR signaling in the VTA provides a plausible explanation for the effectiveness of α_1_-AR antagonists in attenuating CS-induced cocaine seeking (Solecki et al., 2018) as well as CS-induced, but not stress-induced, reinstatement of cocaine seeking (Solecki et al., 2019a). Furthermore, we have previously shown that intra-VTA NA signaling is not engaged in CS-induced food seeking in food-sated animals–a behavioral response to CSs with little salience (Solecki et al., 2018, 2019a).

## Conclusion

Our results suggest a novel functional role of VTA α_1_-AR signaling in the acquisition of Pavlovian association learning in the CPP paradigm. We propose that a salient stimulus such as cocaine, upregulates NAergic activity in the VTA, likely *via* α_1_-ARs expressed on DA neurons. This modulates downstream DA signaling necessary for the encoding of the emotionally arousing events. Accordingly, NA signaling has a facilitating role in the acquisition and/or reconsolidation of emotional memories (Cahill et al., 1994; Ferry et al., 1999; Sara et al., 1999; McGaugh and Roozendaal, 2002; Gelinas and Nguyen, 2005; Milton et al., 2008; Bernardi et al., 2009; Furini et al., 2009; Dȩbiec et al., 2011; Schutsky et al., 2011). Salient stimuli have been shown to upregulate NA system activity, leading to phasic NA release at terminals (Bouret and Richmond, 2009; Bouret et al., 2012; Park et al., 2012). This, at least in the PFC, is thought to be sufficient for salience encoding (Feenstra, 2000; Mingote et al., 2004; Ventura et al., 2007; Puglisi-Allegra and Ventura, 2012). In addition, tonic or slow phasic activity of NA neurons positively correlates with alertness (Aston-Jones and Bloom, 1981; Carter et al., 2010), and NAergic nuclei A1 and A2 are hypothesized to serve as major sensory relays regulating arousal and attention states (Robertson et al., 2013). Similarly, inhibition of VTA DA neuron activity (as expected after intra-VTA α_1_-AR blockade) decreases arousal, even in the face of ethologically relevant, salient stimuli (Eban-Rothschild et al., 2016). Thus, we hypothesize that selective α_1_-AR antagonists in the VTA impair salience detection, reflecting the ability of α_1_-AR signaling in the VTA to modulate alerting and/or orienting functions, originating from bottom-up information processing.

## Data availability statement

The raw data supporting the conclusions of this article will be made available by the authors, without undue reservation.

## Ethics statement

The animal study was reviewed and approved by the 2nd Local Institutional Animal Care and Use Committee, Institute of Pharmacology, Polish Academy of Sciences in Kraków, Poland.

## Author contributions

WS designed and performed the experiments, analyzed the results, performed the histological verifications, and wrote the manuscript. KG and KP performed the surgeries on the animals. KG and AK performed parts of the behavioral experiments and histological verifications. KR-S performed the USVs analysis. JB performed part of FSCV recordings. MK wrote parts of the manuscript. RP provided critical review. All authors contributed to and have approved the final manuscript.

## Supplementary material

Supplementary Figure 1 Localization of histologically verified cannula and FSCV electrode placements. **(A)** Locations of recording electrode in the NAc core (blue) and electrical stimulation and prazosin infusion sites in the VTA (red). **(B)** VTA guide cannula placements in animals from the cocaine-induced place preference experiment (vehicle, low dose and high dose prazosin, respectively). **(C)** VTA guide cannula placements in animals from the cocaine self-administration experiment (vehicle and prazosin infusion sites). **(D)** VTA guide cannula placements in animals in which the effects of prazosin on cocaine-induced USVs were tested (vehicle and prazosin infusion sites). **(E)** VTA guide cannula placements in animals in which CPP in response to intra-VTA prazosin was tested (vehicle, low and high prazosin dose infusion sites, respectively). **(F)** Microinfusion cannula misplacements anterior to the VTA in behavioral experiments measuring effects of intra-VTA α_1_-AR blockade. Drawings are adapted from Paxinos and Watson (2013). All coordinates were obtained from the rat brain atlas by Paxinos and Watson (2013).

Supplementary Figure 2 Effects of intra-VTA micro-infusion of prazosin, a selective α_1_-AR antagonist, on cocaine-induced conditioned place preference (CPP) after one **(A)** or two **(B)** cocaine conditionings. Intra-VTA micro-infusion of prazosin (0.1–1 μg/side) attenuated acquisition of CPP measured as a difference between time spent in cocaine-paired arm during post- and pre-test (**A:** two-way ANOVA *F*_(3, 98)_ = 2.84, *p* < 0.05; **B:** two-way ANOVA *F*_(2, 46)_ = 15.77, *p* < 0.0001). Data are presented as the mean and SEM. ^**^ for *p* < 0.01; ^****^ for *p* < 0.0001.

Supplementary Figure 3 Behavioral effects of intra-VTA micro-infusion of prazosin, a selective α_1_-AR antagonist. **(A)** The experimental time-line and schedule of intra-VTA micro-infusions in CPP paradigm. **(B)** There were no pre-existing differences in the time spent in the prazosin- and vehicle-paired chambers during pre-test between future prazosin (Praz)- and vehicle (Veh)-treated subjects. **(C,D)** Intra-VTA micro-infusion of prazosin (0.5–1 μg/side) did not support acquisition of any conditional responses measured as **(C)** CPP score or **(D)** delta score. **(E,F)** Intra-VTA micro-infusion of prazosin (Praz; 0.5–1 μg/side) did not change the locomotor activity in the open field measured as **(E)** total distance traveled or **(F)** distance traveled over time. Data are presented as individual dots as well as the mean and SEM.
