# Supplementary material for: Alpha1-adrenergic receptor blockade in the ventral tegmental area attenuates acquisition of cocaine-induced pavlovian associative learning
Source: Front Behav Neurosci. 2022 Aug 4;16:969104. doi: 10.3389/fnbeh.2022.969104 (PMC9386374; doi:10.3389/fnbeh.2022.969104)
Supplement: Supplementary file 1 [file Table_1.DOCX]

Table S1. A table listing the factors and levels of ANOVA according to the drug microinfusions and behavioral tests. Conditioned place preference (CPP), open field test (OF), ultrasonic vocalizations (USVs), fixed ratio 1 (FR1), PBS (Veh), prazosin (Praz), saline (sal), cocaine (coc).

| **Test** | **Statistics** | **Treatment** | **Factors** | **Levels** |
| --- | --- | --- | --- | --- |
| CPP (CPP score or delta score); intra-VTA prazosin effects | One-way ANOVA with Newman Keuls *post hoc* | Prazosin | Dose | Veh; Praz 0.5; Praz 1 µg |
| CPP (CPP score or delta score); cocaine dose-response | One-way ANOVA with Newman Keuls *post hoc* | Cocaine | Dose | Sal, coc 15, coc 20 and coc 25 mg/kg |
| Preconditioning in CPP (time spent in conditioning arms during pretest) | Two-way ANOVA with Newman Keuls *post hoc* | Future prazosin | Dose | Veh; Praz 1 µg |
|  |  |  | Chamber | Drug-paired; Saline-paired |
|  |  |  | Interaction | Dose × Chamber |
| Total distance travelled in the OF; intra-VTA prazosin effects | One-way ANOVA with Newman Keuls *post hoc* | Prazosin | Dose | Veh; Praz 0.5; Praz 1 µg |
| OF; distance over time | Two-way repeated-measures ANOVA with Newman Keuls *post hoc* | Prazosin | Dose | Veh; Praz 0.5; Praz 1 µg |
|  |  |  | Time | 5 - 30 min |
|  |  |  | Interaction | Dose × Time |
| Number of cocaine infusions during cocaine self-administration | Two-way repeated-measures ANOVA with Newman Keuls *post hoc* | Future prazosin | Dose | Veh; Praz 0.5; Praz 1 µg |
|  |  |  | Time | 5 - 30 min |
|  |  |  | Interaction | Dose × Time |
| FR1 responding during cocaine self-administration; intra-VTA prazosin effects | Two-way ANOVA with Newman Keuls *post hoc* | Prazosin | Dose | Veh; Praz 1 µg |
|  |  |  | Lever | Active; Inactive |
|  |  |  | Interaction | Dose × Lever |
| FR1 responding during cocaine self-administration; intra-VTA prazosin effects over time | Two-way repeated-measures ANOVA with Newman Keuls *post hoc* | Prazosin | Dose | Veh; Praz 1 µg |
|  |  |  | Time | 5 – 60 min |
|  |  |  | Interaction | Dose × Time |
| FR1 responding during cocaine self-administration; intra-VTA prazosin effects 24 h later | Two-way ANOVA with Newman Keuls *post hoc* | Prazosin | Dose | Veh; Praz 1 µg |
|  |  |  | Lever | Active; Inactive |
|  |  |  | Interaction | Dose × Lever |
| Acquisition of FR1 responding during cocaine self-administration | Three-way repeated-measures ANOVA with Newman Keuls *post hoc* | Future prazosin | Dose | Veh; Praz 1 µg |
|  |  |  | Lever | Active; inactive |
|  |  |  | Time | Day 1-8 |
|  |  |  | Interaction | Dose × Lever |
|  |  |  |  | Dose × Time |
|  |  |  |  | Dose × Time × Lever |
| Cocaine-evoked USVs; intra-VTA prazosin effects on USVs category | Two-way ANOVA with Newman Keuls *post hoc* | Prazosin | Dose | Veh; Praz 1 µg |
|  |  |  | USVs category | Complex; Upward ramp; Downward ramp; Flat; Short; Split; Step-up; Step-down; Multi-step; Trill; Flat-trill combination; Inverted U; Composite; Alarm calls |
|  |  |  | Interaction | Dose × USVs category |
| Cocaine-evoked USVs; intra-VTA prazosin effects | Two-way ANOVA with Newman Keuls *post hoc* | Prazosin | Praz treatment | Veh; Praz 1 µg |
|  |  |  | Cocaine dose | sal; 10; 20 mg/kg |
|  |  |  | Interaction | Praz treatment × Cocaine dose |
| Phasic DA release in the NAc; intra-VTA prazosin effects | Two-way repeated-measures ANOVA with Newman Keuls *post hoc* | Prazosin | Dose | Veh; Praz 1 µg |
|  |  |  | Time | 3 – 15 min |
|  |  |  | Interaction | Dose × Time |
