# Supplementary figures and images for: Alpha1-adrenergic receptor blockade in the ventral tegmental area attenuates acquisition of cocaine-induced pavlovian associative learning
Source: Front Behav Neurosci. 2022 Aug 4;16:969104. doi: 10.3389/fnbeh.2022.969104 (PMC9386374; doi:10.3389/fnbeh.2022.969104)

A

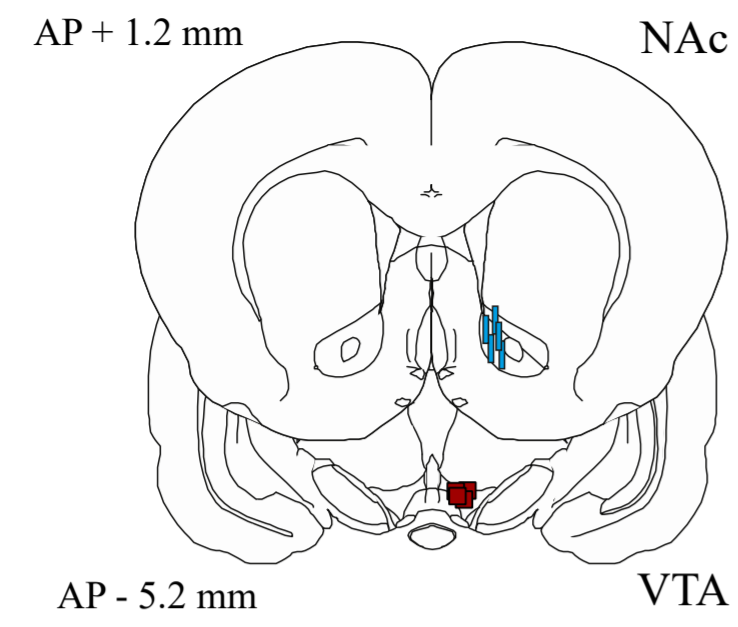

B

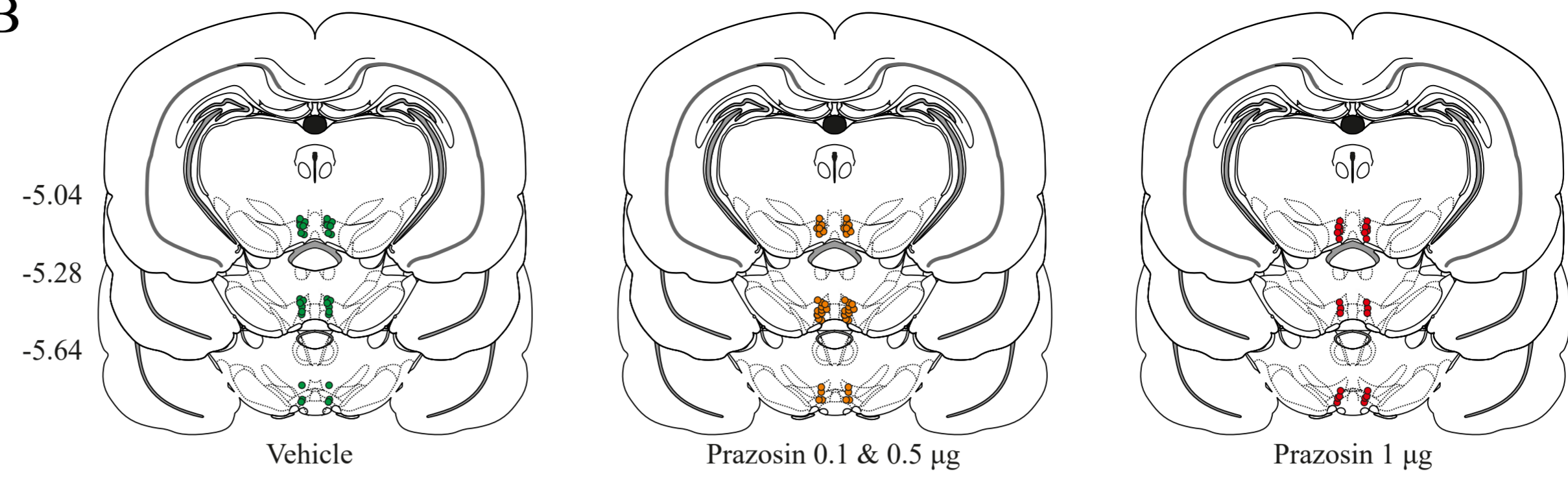

C

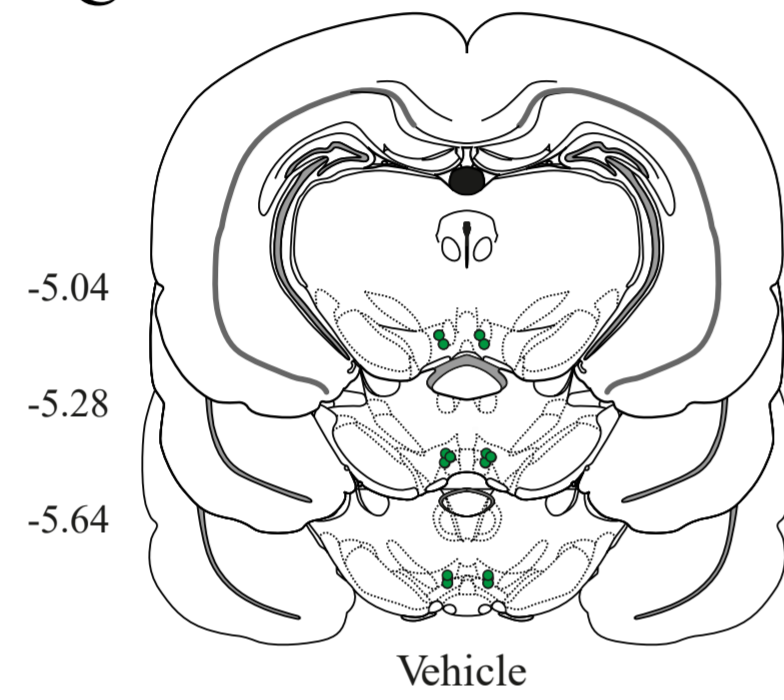

D

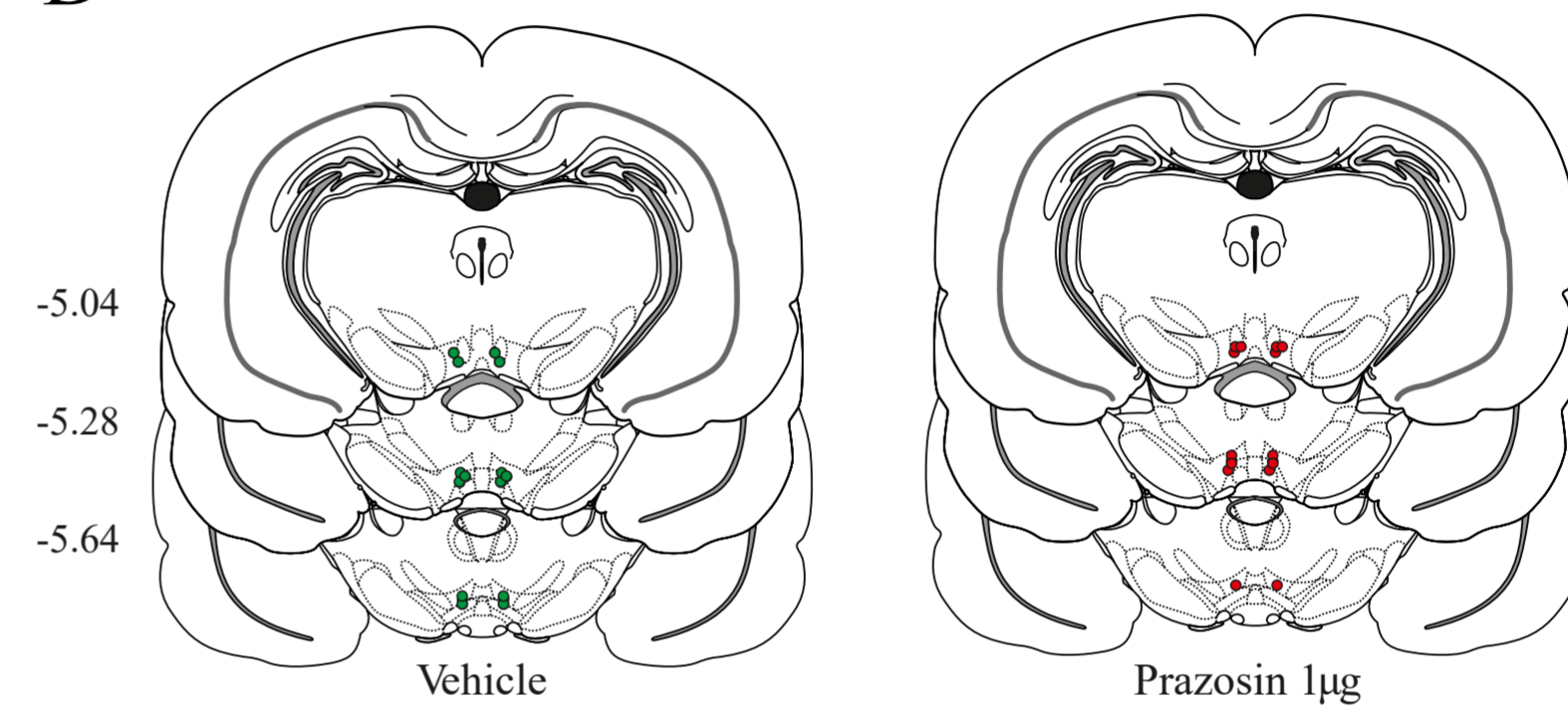

E

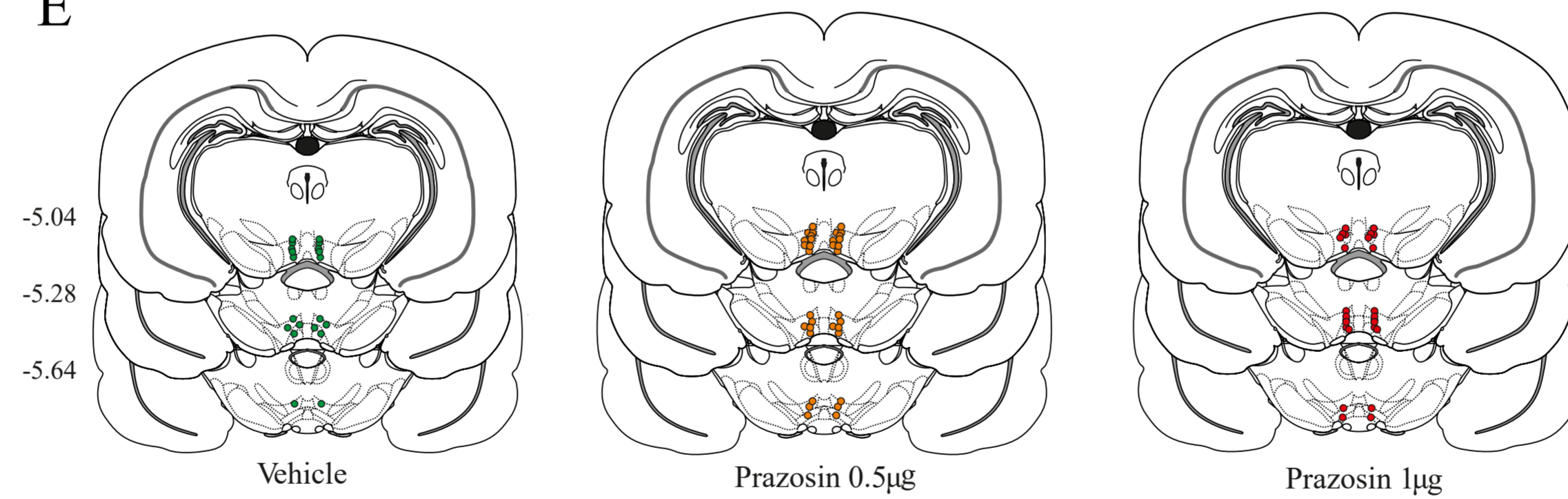

F

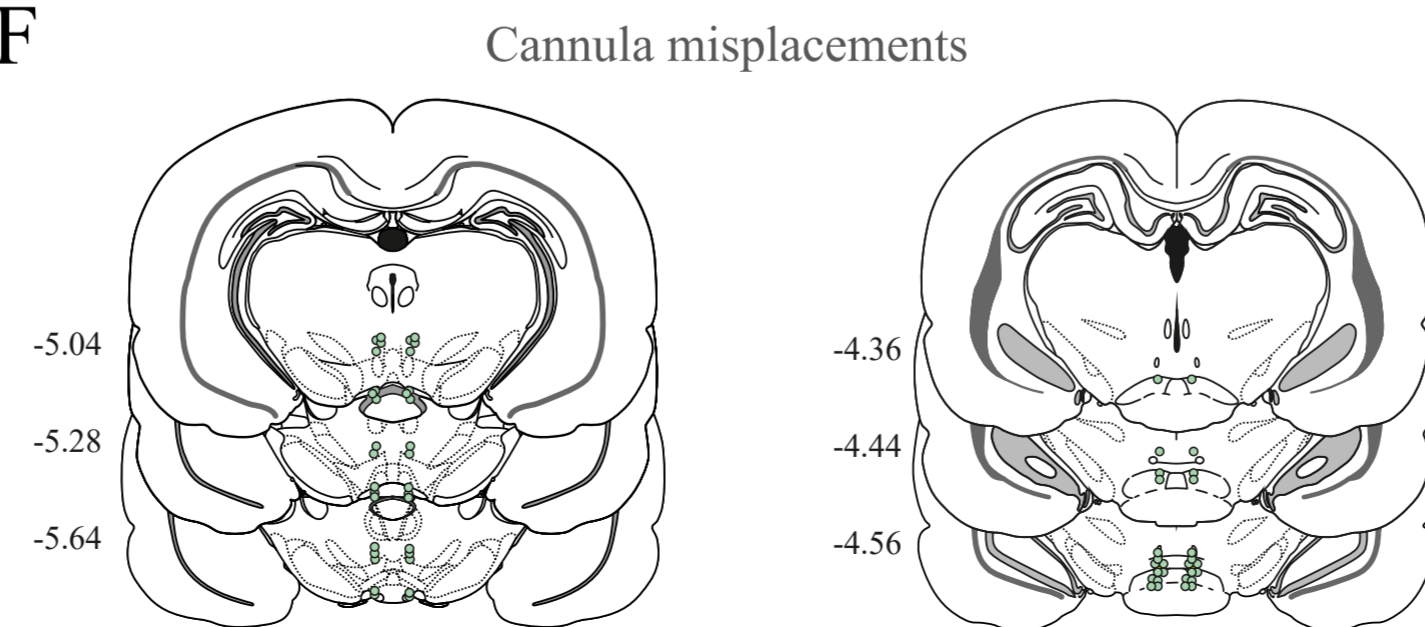

Supplement: Supplementary Figure 1 — Localization of histologically verified cannula and FSCV electrode placements. (A) Locations of recording electrode in the NAc core (blue) and electrical stimulation and prazosin infusion sites in the VTA (red). (B) VTA guide cannula placements in animals from the cocaine-induced place preference experiment (vehicle, low dose and high dose prazosin, respectively). (C) VTA guide cannula placements in animals from the cocaine self-administration experiment (vehicle and prazosin infusion sites). (D) VTA guide cannula placements in animals in which the effects of prazosin on cocaine-induced USVs were tested (vehicle and prazosin infusion sites). (E) VTA guide cannula placements in animals in which CPP in response to intra-VTA prazosin was tested (vehicle, low and high prazosin dose infusion sites, respectively). (F) Microinfusion cannula misplacements anterior to the VTA in behavioral experiments measuring effects of intra-VTA α1-AR blockade. Drawings are adapted from Paxinos and Watson (2013). All coordinates were obtained from the rat brain atlas by Paxinos and Watson (2013). [file Image_1.pdf]

A

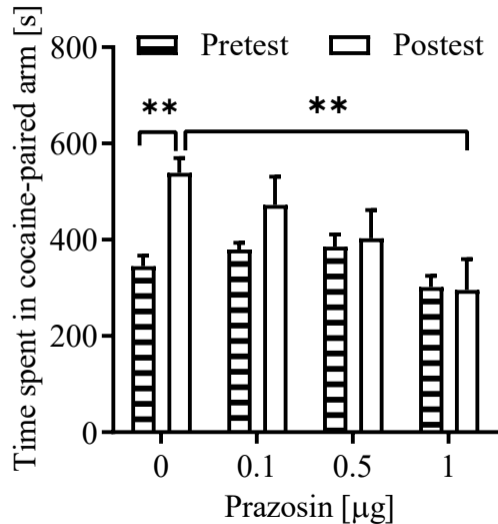

B

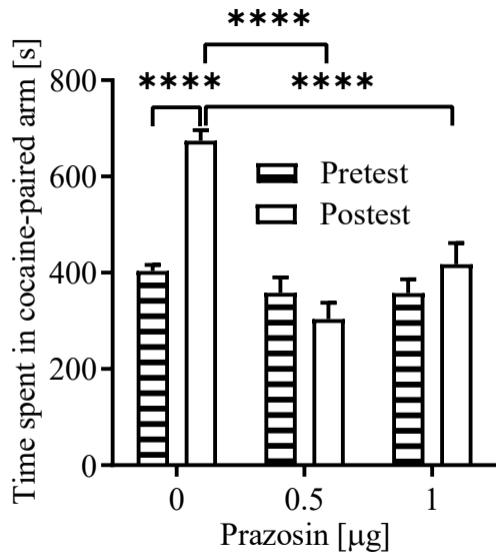

Supplement: Supplementary Figure 2 — Effects of intra-VTA micro-infusion of prazosin, a selective α1-AR antagonist, on cocaine-induced conditioned place preference (CPP) after one (A) or two (B) cocaine conditionings. Intra-VTA micro-infusion of prazosin (0.1–1 μg/side) attenuated acquisition of CPP measured as a difference between time spent in cocaine-paired arm during post- and pre-test (A: two-way ANOVA F(3, 98) = 2.84, p < 0.05; B: two-way ANOVA F(2, 46) = 15.77, p < 0.0001). Data are presented as the mean and SEM. ** for p < 0.01; **** for p < 0.0001. [file Image_2.pdf]

A

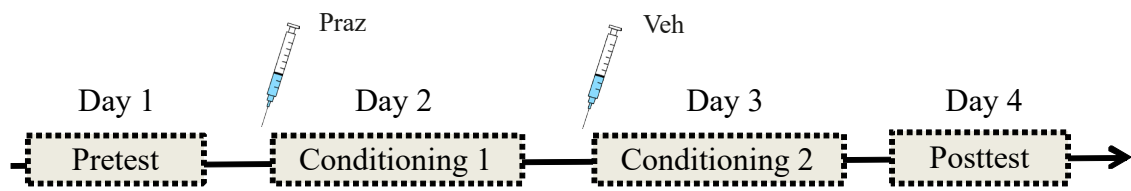

B

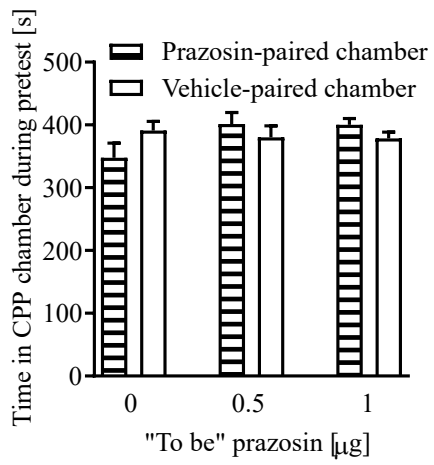

C

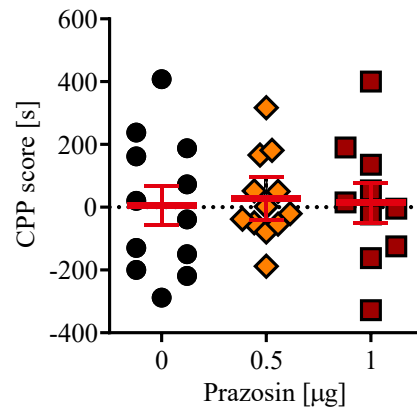

D

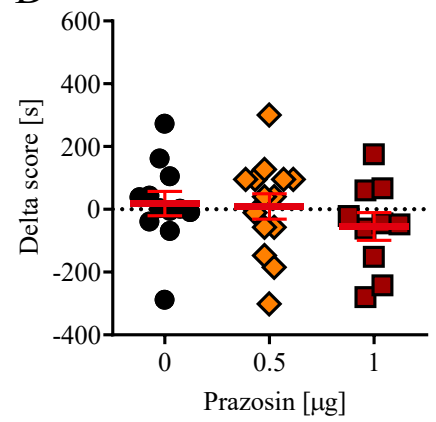

E

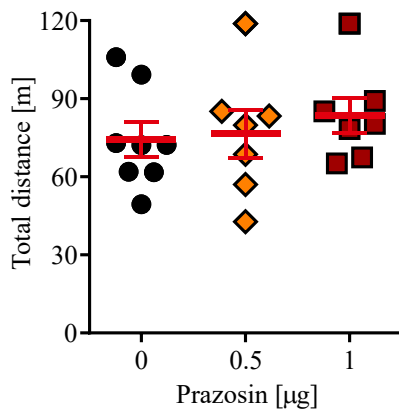

F

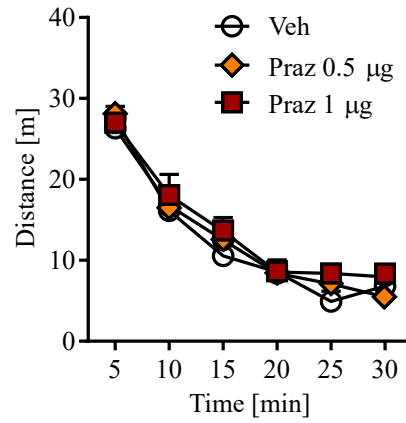

Supplement: Supplementary Figure 3 — Behavioral effects of intra-VTA micro-infusion of prazosin, a selective α1-AR antagonist. (A) The experimental time-line and schedule of intra-VTA micro-infusions in CPP paradigm. (B) There were no pre-existing differences in the time spent in the prazosin- and vehicle-paired chambers during pre-test between future prazosin (Praz)- and vehicle (Veh)-treated subjects. (C,D) Intra-VTA micro-infusion of prazosin (0.5–1 μg/side) did not support acquisition of any conditional responses measured as (C) CPP score or (D) delta score. (E,F) Intra-VTA micro-infusion of prazosin (Praz; 0.5–1 μg/side) did not change the locomotor activity in the open field measured as (E) total distance traveled or (F) distance traveled over time. Data are presented as individual dots as well as the mean and SEM. [file Image_3.pdf]
